# Tumor cell-intrinsic phenotypic plasticity facilitates adaptive cellular reprogramming driving acquired drug resistance

**DOI:** 10.1007/s12079-017-0435-1

**Published:** 2017-11-30

**Authors:** Heinz Hammerlindl, Helmut Schaider

**Affiliations:** The University of Queensland Diamantina Institute, Translational Research Institute, The University of Queensland, 37 Kent Street, Brisbane, QLD 4102 Australia

**Keywords:** Drug resistance, Adaptive drug tolerance, Phenotypic plasticity, Epigenetic remodeling, Drug tolerant persisters, Induced drug tolerance, Transcriptional reprograming, Targeted inhibitors, Stress response, Slow cycling cancer cells

## Abstract

The enthusiasm about successful novel therapeutic strategies in cancer is often quickly dampened by the development of drug resistance. This is true for targeted therapies using tyrosine kinase inhibitors for *EGFR* or *BRAF* mutant cancers, but is also an increasingly recognized problem for immunotherapies. One of the major obstacles of successful cancer therapy is tumor heterogeneity of genotypic and phenotypic features. Historically, drivers for drug resistance have been suspected and found on the genetic level, with mutations either being pre-existing in a subset of cancer cells or emerging de novo to mediate drug resistance. In contrast to that, our group and others identified a non-mutational adaptive response, resulting in a reversible, drug tolerant, slow cycling phenotype that precedes the emergence of permanent drug resistance and is triggered by prolonged drug exposure. More recently, studies described the importance of initially reversible transcriptional reprogramming for the development of acquired drug resistance, identified factors important for the survival of the slow cycling phenotype and investigated the relationship of mutational and non-mutational resistance mechanisms. However, the connection and relative importance of mutational and adaptive drug resistance in relation to the in vitro models at hand and the clinically observed response patterns remains poorly defined. In this review we focus on adaptive intrinsic phenotypic plasticity in cancer cells that leads to the drug tolerant slow cycling state, which eventually transitions to permanent resistance, and propose a general model based on current literature, to describe the development of acquired drug resistance.

## Introduction

Human cancers, which are generally assumed to originate from a single cell (Boyd et al. 2012; Rycaj and Tang 2015), display surprising heterogeneity of phenotypic and genotypic features (Marusyk and Polyak 2010). This heterogeneity has long been known (Fidler 1978) and was extensively studied in the following decades. Since then several models have been developed to describe intratumoral heterogeneity. The two most appealing ones are the classical Darwinian-like clonal evolution, which describes the selection of sub clones with equal tumorigenic potential and the cancer stem cell (CSC) model constituting a hierarchical organization within the tumor were a small population of CSCs gives rise to non-tumorigenic progenitors establishing the tumor with all the heterogeneous phenotypes (Cabrera et al. 2015; Marusyk and Polyak 2010; Vlashi and Pajonk 2015). Even though there is convincing evidence for either model (reviewed in (Apostoli and Ailles 2016)), the observed tumor heterogeneity is complex and it appears highly likely that both models are not mutually exclusive but rather co-exist (Cabrera et al. 2015; Shackleton et al. 2009). An additional layer of complexity is added by phenotypic plasticity, which describes changes of cellular states either stochastically or in response to an external stimulus (Marusyk et al. 2012). Recent studies showed bidirectional interconversions between stem like and non-stem like states (Chaffer et al. 2011; Chaffer et al. 2013; Gupta et al. 2011) which is incompatible with the hierarchical unidirectional CSC model suggesting a key role of phenotypic plasticity for tumor heterogeneity.

Since the declaration of the “War on Cancer” (Hanahan 2014), tremendous research efforts combined with technical advances, like high throughput sequencing, resulted in extraordinary progress to our understanding of cancer pathogenesis. Identifying “driver” mutations in cancer allowed for the design and development of targeted drugs that specifically inhibited mutation induced pathways. A prime example are inhibitors targeting the BRAF kinase that is mutated in approximately 50% of cutaneous melanomas but is also found in colorectal cancer, non–small-cell lung cancer, papillary thyroid cancer, diffuse gliomas and cholangiocarcinoma (Hyman et al. 2015). These drugs show a remarkable clinical response compared to conventional chemotherapy (Ugurel et al. 2016) and represent one of the most distinct milestones in the “War on Cancer”.

Unfortunately, responses to targeted therapy are often short lived (Holohan et al. 2013) and immunotherapies as well lead to the development of drug resistance (Sharma et al. 2017). One of the major challenges for successful cancer therapy is the aforementioned tumor heterogeneity. Melanomas for example, show substantial heterogeneity at the genetic as well as the biological level. Genetic heterogeneity includes the presence of *BRAF* ^V600E^/*NRAS* ^wt^ and *BRA*F^wt^/*NRAS* ^Q16R^ in the same lesion (Sensi et al. 2006), heterogeneity of *BRAF* ^V600E^ and *BRAF* ^wt^ within a primary melanoma (Lin et al. 2011), or between primary and metastatic melanomas (Yancovitz et al. 2012). Examples for biological heterogeneity include switching between proliferative and invasive states (Hoek et al. 2008), variable expression of tumor-associated antigens (Slingluff et al. 2000) as well as dynamic expression of CSC markers including *CD133* (Shackleton et al. 2009), *ABCB5*, *NGFR* (Quintana et al. 2010) or *KDM5B* (Roesch et al. 2010). Considering the dynamic expression pattern of these CSC markers, combined with the fact that literally every melanoma cell has tumor initiating potential (Quintana et al. 2010), evidence supports a prominent role for phenotypic plasticity as source for tumor heterogeneity in melanoma. Similar observations were made in other solid cancer types (Gay et al. 2016; McGranahan and Swanton 2017). The consequences of the highly heterogeneous nature of tumors are reflected in the clinical presentation of the therapeutic response and represent a monumental challenge for clinical success of cancer treatment strategies.

Hereafter, we will discuss the origin of drug resistance with a focus on adaptive phenotypic plasticity and propose a general model based on current literature, in an effort to describe the response of cancer cells to chronic drug exposure.

### Intrinsic drug resistance

**Fig. 1:**
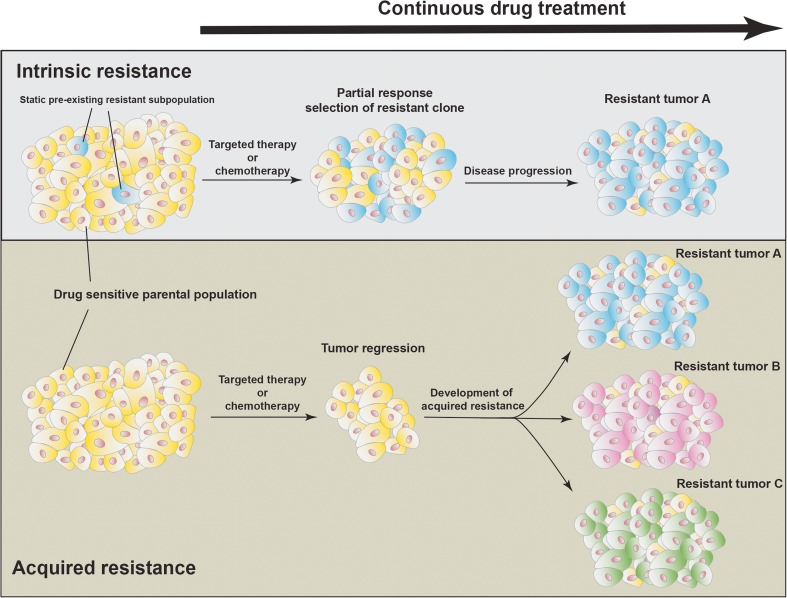
**Acquired and intrinsic drug resistance.** Static, pre-existing subpopulations within a tumor can mediate intrinsic drug resistance. These subpopulations often harbor resistance mediating mutations, which quickly become the predominant population resulting in minor response and rapid progression. In contrast, drug sensitive tumors show a rapid regression in response to anti-cancer drugs followed by the development of a variety of acquired resistance mechanisms (resistance A, B or C)

Intrinsic resistance is characterized as non-responsiveness towards a specific therapy or the rapid progression despite therapy, which is caused by resistance mediating, pre-existing mutations or other cellular features that are often present in subpopulations of the tumor (Fig. 1). Such intrinsic resistance mechanisms are present in a subset of patients with a well-defined mutational background. In melanoma, 48–59% of tumors harboring the *BRAF* ^*V600E*^ mutation show a clinical response to BRAF inhibition (Hauschild et al. 2012). In contrast, *BRAF* mutant colorectal cancers that count for approximately 10% of all cases, show only a marginal response rate of 5% (Prahallad et al. 2012), suggesting that colorectal cancers have an intrinsic resistance mechanism that appears to be absent or less common in melanoma. Prahallad et al. investigated this astounding difference using shRNA mediated knockdown to screen for the involvement of 518 human kinases and 17 additional kinase-related genes and found that knockdown of *EGFR* sensitized *BRAF* mutant colorectal cancer cells to BRAF inhibition. Mechanistically, BRAF inhibition resulted in decreased activation of CDC25C, a phosphatase involved in dephosphorylation and inactivation of EGFR, followed by rapid activation of EGFR and its downstream target AKT. Accordingly, combined BRAF and EGFR inhibition showed synergistic efficiency in colorectal cancer cells in vitro and in vivo (Prahallad et al. 2012). Melanomas are derived from the neural crest and therefore have low endogenous EGFR expression, which explains the stunning intrinsic difference in drug sensitivity of two cancer types that are driven by the same mutation (Prahallad et al. 2012). *EGFR* itself is mutated in approximately 20% of all non-small-cell lung cancers (NSCLCs), with significantly increased prevalence in patients of Asian ethnicity (Wang et al. 2016). Similar to mutant *BRAF*, several drugs have been developed to target mutant *EGFR*, with response rates over 70% in patients with disease driven by activating *EGFR* mutations (Mok et al. 2009). However the genetic landscape of *EGFR* mutations is more complex. The majority of tumors (>90%) show an in-frame deletion in exon 19 or a L858R substitution in exon 21 that result in constitutively active EGFR signaling and can be inhibited by first-generation EGFR inhibitors like erlotinib or gefitinib (Mok et al. 2009). Beside these drug vulnerable mutations, specific insertion mutations in exon 20*,* which represent 5–10% of all *EGFR* mutations, have been shown to be non-responsive to first-generation EGFR inhibitors (Greulich et al. 2005; Naidoo et al. 2015). Therefore, *EGFR* is a prime example how different mutations in the same gene that all result in constitutive pathway activation can confer intrinsic resistance to specific small molecule inhibitors. In addition to the common activating *EGFR* mutations, the substitution of methionine for threonine at position 790 of the *EGFR* gene has been found to confer resistance to first generation EGFR inhibitors by preventing the binding of the inhibitor to the ATP binding pocket (Wang et al. 2016). This mutation is commonly found in patients with acquired resistance to first generation EGFR inhibitors (Yu et al. 2013) but can also be found in treatment naïve tumors. The detection frequency of pre-existing *EGFR* ^*T790M*^ is highly variable, depending on the detection method (2–80%) (Wang et al. 2016) and is associated with decreased progression free survival and overall survival (Lee et al. 2014), suggesting that pre-existing *EGFR* ^*T790M*^ is an intrinsic resistance mechanism to EGFR inhibition in *EGFR* mutant NSCLC. The third generation of EGFR inhibitors like osimertinib, an oral, irreversible inhibitor specifically designed to target *EGFR* ^*L858R*^, *EGFR* ^*del19*^, and *EGFR* ^*T790M*^ (Liao et al. 2016), showed a clinical response of 71% in patients with *EGFR* ^*T790M*^ positive lung cancer with significantly improved progression-free survival compared to patients receiving platinum therapy plus pemetrexed (Mok et al. 2017). Despite this success, a significant number of patients is not responding to these third generation inhibitors and resistance has been linked to yet another *EGFR* mutation (*EGFR* ^*C797S*^) (Thress et al. 2015).

### Acquired drug resistance

In contrast to intrinsic resistance that prevents any meaningful clinical response to anti-cancer therapy, acquired resistance develops after a significant initial response over the course of several month. In many cases, tumors that progressed after an initial success acquired new resistance mediating mutations. One such example is the previously mentioned *EGFR* ^*T790M*^ mutation, which can be found in approximately 60% of patients that progressed after therapy with first generation EGFR inhibitors (Yu et al. 2013). To determine if the high prevalence of *EGFR* ^*T790M*^ in tumors that relapsed after successful first generation EGFR inhibitor therapy is strictly the result of selection of pre-existing sub clones Hata et al. generated multiple resistant clones of PC9 cells in parallel, using escalating concentrations of the EGFR inhibitor, gefitinib (Hata et al. 2016). This method resulted in an unexpected variance of time to resistance, with a few clones regaining rapid proliferation after 2–3 weeks of drug exposure, while others required up to 40 weeks to develop resistance. Using lentiviral Clontracer barcodes, Hata et al. was able to show that the early resistant clones derived from pre-existing subpopulations that are present at frequencies below the detection limit of standard next-generation sequencing or droplet digital PCR (Hata et al. 2016). Accordingly, only 90 of 1260 tested cell pools showed early resistance mediated by pre-existing subpopulations, while the majority resembled a slow cycling drug tolerant state that was previously linked to chromatin remodeling (Hata et al. 2016; Sharma et al. 2010). Continuous treatment of cell pools without signs of early resistance for up to 47 weeks resulted in the emergence of *EGFR* ^*T790M*^ positive clones in approximately 30% of the tested cell pools, with undisclosed resistance mechanisms in the remaining samples. Transcriptional profiling revealed that late *EGFR* ^*T790M*^ positive clones shared features of the slow cycling drug tolerant state while early *EGFR* ^*T790M*^ positive clones clustered with parental cells (Hata et al. 2016), suggesting that de novo emergence of *EGFR* ^*T790M*^ from a slow cycling drug tolerant state is one of multiple mechanisms of acquired resistance to first generation EGFR inhibitors. Similarly, it recently has been shown that unsupervised clustering of transcriptional profiles including the top 3000 most variant genes of melanoma patient derived cell lines that developed resistance after long term exposure to BRAF or BRAF/MEK inhibitors, distinguished two different clusters (Song et al. 2017). One cluster included resistant cell lines with specific drivers of MAPK reactivation/addiction, like *BRAF* splicing, *BRAF* amplification or *NRAS* mutations that were previously linked to BRAF inhibitor resistance (Bhang et al. 2015; Shi et al. 2014) and clustered together with parental transcriptional profiles. The second cluster displayed transcriptomic reprogramming away from MAPK dependent and therefore BRAF and BRAF/MEK inhibitor sensitive parental cells (Song et al. 2017). Interestingly, both resistant cell types emerged after an initial transition into a slow cycling state and the trajectory of the transcriptomic profiles of these slow cycling cells were found to be similar to the resistant cells that displayed cellular reprogramming. This suggests that drug exposure initially results in transient cellular reprogramming that decreases MAPK dependency, which can be followed by either re-establishment of hyperactivated MAPK signaling as mediated by genetic alterations and overexpression of components of the MAPK pathway, or aggravating and persistent cellular reprogramming away from an MAPK signaling dependent phenotype. Importantly, transcriptomic profiles of the majority of on treatment, but not progressive disease patient tumors showed similar trajectories, suggesting cellular reprogramming in patients (Song et al. 2017). Taken together, the studies performed by Hata et al. and Song et al. convincingly show that mutation induced drug resistance can be either intrinsic (pre-existing) or acquired (de novo mutations) and highlight the presence of an cancer and drug type independent early slow cycling drug tolerant state that undergoes cellular reprogramming to survive initial drug exposure, which then give rise to mutational and non-mutational drug resistance (Fig. 1).

### The slow cycling phenotype

Phenotypic switching between a drug sensitive proliferative state and a drug tolerant slow growing state in a genetically homogeneous population, which is associated with increased drug resistance was described in bacteria more than a decade ago (Balaban et al. 2004) and has yet to be fully understood and overcome (Holden 2015). Interestingly, it was shown that bacteria in the slow growing state that persisted after antibiotic treatment could spontaneously switch to a proliferative state upon drug removal and give rise to a drug sensitive population (Balaban et al. 2004), indicating that these sub clones did not acquire resistance mediating mutations. Similar to this, Sharma et al. described a reversible, slow growing, mainly G1 arrested subpopulation in cancer cells that persists after exposure to anti-cancer drugs at concentrations 100-fold greater than the IC_50_ (Sharma et al. 2010). The slow growing population was able to re-establish the drug sensitive heterogeneous parental population upon drug withdrawal and single cell derived clones showed a similar number of slow growing drug tolerant cells once challenged with anti-cancer drugs (Sharma et al. 2010). This suggests that the slow cycling phenotype is not a static, pre-existing subpopulation but rather a dynamically regulated phenotype that is stochastically occurring within cancer cells. Supporting this hypothesis, Shaffer et al. recently identified rare cell variability that conferred BRAF inhibitor resistance (Shaffer et al. 2017). They analyzed transcriptomics profiles of drug sensitive and drug resistant cell populations utilizing high-throughput single-molecule RNA fluorescence in situ hybridization (FISH) to identify pre-existing single cells with high expression levels of individual resistance markers, including *WNT5A*, *AXL*, *EGFR*, *PDGFRB*, *NGFR* and *JUN.* Expression levels of these marker genes showed high variability in single cells but were found to have a high degree of co-expression (Shaffer et al. 2017). Isolated cells with increased expression of either *EGFR*, *NGFR* or *AXL* were more likely to become resistant upon drug exposure compared to unsorted parental cells. Accordingly, expression of multiple markers further increased the tendency to become resistant and the cells gradually reprogrammed their transcriptional profile to resemble the stably resistant state (Shaffer et al. 2017). This is in line with Song et al. (Song et al. 2017) and suggests that dynamic drug induced cellular reprogramming is a key mechanism of acquired drug resistance. In accordance with the dynamic drug induced cellular reprograming found in melanoma and lung cancer, we identified that chronic, sub lethal drug exposure triggers a cellular response leading to a slow cycling, multidrug tolerant phenotype (Ravindran Menon et al. 2015). These so called induced drug-tolerant cells (IDTCs) appear to be the result of cellular reprogramming and not the selection of a pre-existing subpopulation, as the majority of cells survived the drug treatment and adopted the slow cycling, mainly G1 arrested phenotype that is reversible upon drug withdrawal. IDTCs are characterized by increased expression of melanoma stem cell markers like *NGFR*, *SOX10* and *CD44* as well as increased expression of drug efflux genes including *ABCB5*, *ABCA5*, *ABCB8* and *ABCB4*. Accordingly, IDTCs were found to be multi-drug resistant as neither increased concentrations of BRAF inhibitor, the MEK inhibitor GSK1120212 nor cisplatin showed any significant effect on these slow cycling cells (Ravindran Menon et al. 2015). The phenotypic switch displayed profound alterations of histone methylation patterns. We found that IDTCs showed decreased H3K4me3 and H3K27me3 as well as increased H3K9me3, which was accompanied by increased expression of histone modifying enzymes including the H3K27 specific demethylases KDM6A, KDM6B and the H3K4 specific demethylases KDM1B, KDM5A and KDM5B (Ravindran Menon et al. 2015). Increased expression of KDM5A (Sharma et al. 2010) and KDM5B (Roesch et al. 2013) have previously been found to be functionally important markers of slow cycling cancer cells that are enriched upon drug exposure, whereas the pre-existing KDM5B^high^ subpopulation was also found to be essential for continuous tumor growth in melanoma (Roesch et al. 2010). Loss of H3K4me3 and increase of H3K9me3 has recently been described in drug induced slow cycling *EGFR* mutant non-small-cell lung carcinoma and other cancer types (Guler et al. 2017). Specifically, multiple H3K9me3 specific histone methyltransferases, including *SETDB1* and *EHMT2* are upregulated and important for the survival of slow cycling drug tolerant cells. In addition, key factors for H3K9me3 mediated heterochromatin formation like HP1γ, ATRX and H3.3 are involved in the formation of the slow cycling drug tolerant state and RNA sequencing revealed an increase in IFN response/antiviral defense genes (Guler et al. 2017). Accordingly, a global increase in repressive chromatin was found in repetitive regions of the genome while IFN responsive genes showed increased chromatin accessibility. Mechanistically, H3K9me3-mediated heterochromatin formation suppressed the expression of LINE-1 elements, which was reversed by HDAC inhibition and contributed to HDAC inhibitor mediated ablation of the slow cycling drug tolerant cell population (Guler et al. 2017). It is noteworthy that our studies showed that short term HDAC inhibitor treatment as used by Guler et al. reversed the histone methylation pattern in slow cycling drug tolerant cells from the characteristic H3K4me3^low^/H3K9me3^high^ to a H3K4me3^high^/H3K9me3^low^ pattern (Ravindran Menon et al. 2015), which is in line with the previously described de-repression of H3K9me3 suppressed LINE-1 elements. However, this was only transient and continuous exposure to BRAF inhibitors, the drug used to trigger the transition into the slow cycling state, in combination with the HDAC inhibitor TSA resulted in the re-establishment of the H3K4me3^low^/H3K9me3^high^ histone methylation pattern characteristic for the slow cycling drug tolerant phenotype (Ravindran Menon et al. 2015). In general, we found that strategies to eradicate slow cycling subpopulations that were identified based on the expression of KDM5A (Sharma et al. 2010) and KDM5B (Roesch et al. 2013) were mostly unsuccessful in our IDTC cells as they only induced a minor growth deficiency (Ravindran Menon et al. 2015). Similar to what was seen for the HDAC inhibitors, continuous co-treatment of BRAF inhibitor derived IDTCs with either an MEK or AKT inhibitor initially suppressed the respective target pathway followed by a gradual re-wiring to re-establish pathway activation (Ravindran Menon et al. 2015). It is possible that slow cycling cells selected based on expression of specific markers like KDM5B represent a subtype of the slow cycling phenotype with specific vulnerabilities to which the bulk of the slow growing cells can further adjust. This hypothesis is supported by the high variability of the transcriptomic profiles of slow cycling drug tolerant cells that gradually reprogram towards the resistant phenotype (Shaffer et al. 2017; Song et al. 2017). Because of this, vulnerabilities of the drug induced slow cycling phenotype could change over time, which has to be considered when comparing different studies and during the development of therapeutic strategies. Furthermore, the very high initial drug concentration to generate slow cycling drug tolerant cancer cells chosen in some studies results in a very small number of surviving cells (Sharma et al. 2010). If such a strategy, which might be clinically not relevant, is employed in combination with treatments aimed to target the slow cycling phenotype, the number of remaining slow cycling cells that further adapts to the new challenge might be too low to perceive within the experimental timeframe.

The histone methylation patterns of the slow cycling drug tolerant state described by Guler et al. is similar to our IDTC state in regards to H3K4me3 and H3K9me3, but it differs from the observed H3K27me3 levels. Guler et al. found increased H3K27me3 levels in their drug tolerant persister population (Guler et al. 2017), while IDTCs showed a global decrease of H3K27me3 after 12 days of drug exposure (Ravindran Menon et al. 2015). In comparison, glioblastoma stem cells (GSC) exposed to targeted kinase inhibitors also undergo a reversible transition into a slow-cycling, persistent state (Liau et al. 2017). This state is characterized by global loss and redistribution of H3K27me3 as well as increased H3K27ac at enhancer-like elements and increased Notch pathway activity and dependency. Accordingly, slow cycling GSCs showed increased expression of H3K27 demethylases KDM6A and KDM6B, which were found to be essential for the emergence of the slow cycling phenotype (Liau et al. 2017) and are also up regulated in our IDTC cells (Ravindran Menon et al. 2015). In line with these observations, slow cycling drug tolerant melanoma cell lines and on treatment tumors undergo a mesenchymal-invasive-angiogenic switch that is manifested by differential H3K27ac and CpG specific hyper- and hypo-methylation (Song et al. 2017). While a conclusive explanation for the discrepancy of H3K27me3 levels in the different studies of drug induced slow cycling cells is difficult, the gradual cellular reprogramming discussed earlier could be involved. Treatment durations and drug concentrations to establish the slow cycling phenotype are different in individual studies, which opens the possibility that loss of H3K4me3 and gain of H3K9me3 are coordinated events while H3K27me3 remodeling is a separate step in the reprogramming process.

### Escaping the slow cycling state by cellular reprogramming to regain proliferation

While evidence for the importance of a therapy induced, reversible, slow cycling, drug tolerant phenotype is accumulating and is being increasingly accepted, how stably resistant cancer cells can emerge from this slow cycling phenotype and details how cancer cells escape the drug induced slow cycling state remain enigmatic. In the work that first identified a epigenetically driven drug induced slow cycling state, Sharma et al. also noted that slow cycling drug tolerant persisters (DTP) will eventually re-enter the cell cycle and regain proliferation following continuous drug exposure (Sharma et al. 2010). These cells showed differential expression of cell surface markers, including CD133 and CD24 compared to DTPs but still regained drug sensitivity upon drug withdrawal (Sharma et al. 2010). In comparison, continuous culturing (6–8 month) of isolated cell clusters that emerged from slow cycling cells generated using the same protocol established by Sharma et al. showed a high diversity of resistance mechanisms and were non- or only partially reversible (Ramirez et al. 2016), suggesting that cells that escape the drug induced slow cycling state have a distinct phenotype that undergoes further alterations to stabilize a range of resistance mechanisms. The hypothesis that re-proliferating drug tolerant colonies present a distinct phenotype are supported by previous findings of Song et al. who shows that BRAF or BRAF/MEK inhibitor exposed cells that escaped the initial slow cycling state have a distinct morphology and transcriptional profile compared to the slow cycling state, whereas the transcriptional profile showed a trajectory towards the MAPK-redundancy resistance type (Song et al. 2017). Further evidence is presented by Shaffer et al. who observed the emergence of drug resistant proliferating colonies from the initially slow cycling cell population during cronic drug exposure, which was directly linked to the dynamic existence of cells expressing a number of resistance marker genes (Shaffer et al. 2017). They utilized a genome-wide assay for transposase-accessible chromatin (ATAC-seq) to map putative transcription factor binding sites and found a global loss of accessible chromatin within the first week of drug treatment (Shaffer et al. 2017). The observed loss of binding sites was mainly attributed to loss of SOX10 binding, suggesting that drug exposure results in a de-differentiated state (Shaffer et al. 2017). This is in line with our observation that IDTCs are characterized by increased CD271 (Ravindran Menon et al. 2015), which is a marker for a de-differentiated state in melanoma (Beretti et al. 2015). This initial loss of accesible chromatin was followed by a gain of transcription factor binding sites after 4 weeks of continuous exposure to BRAF inhibitors, which coincides with the emergnce of drug resistant proliferating colonies. TEAD and AP-1 signaling pathways were among the most highly activated (Shaffer et al. 2017), which was previously associated with increased resistance to MAPK pathway inhibition of an invasive cell state in melanoma (Verfaillie et al. 2015). This phenotype shares similarities to the transcriptional profiles of resistant cells that underwent transcriptomic reprogramming away from MAPK dependence and on-treatment residual melanoma in patients described by Song et al. (Song et al. 2017), suggesting that this in vitro phenotype is also present in patients undergoing cancer therapy.

### Conclusion and future directions

**Fig. 2:**
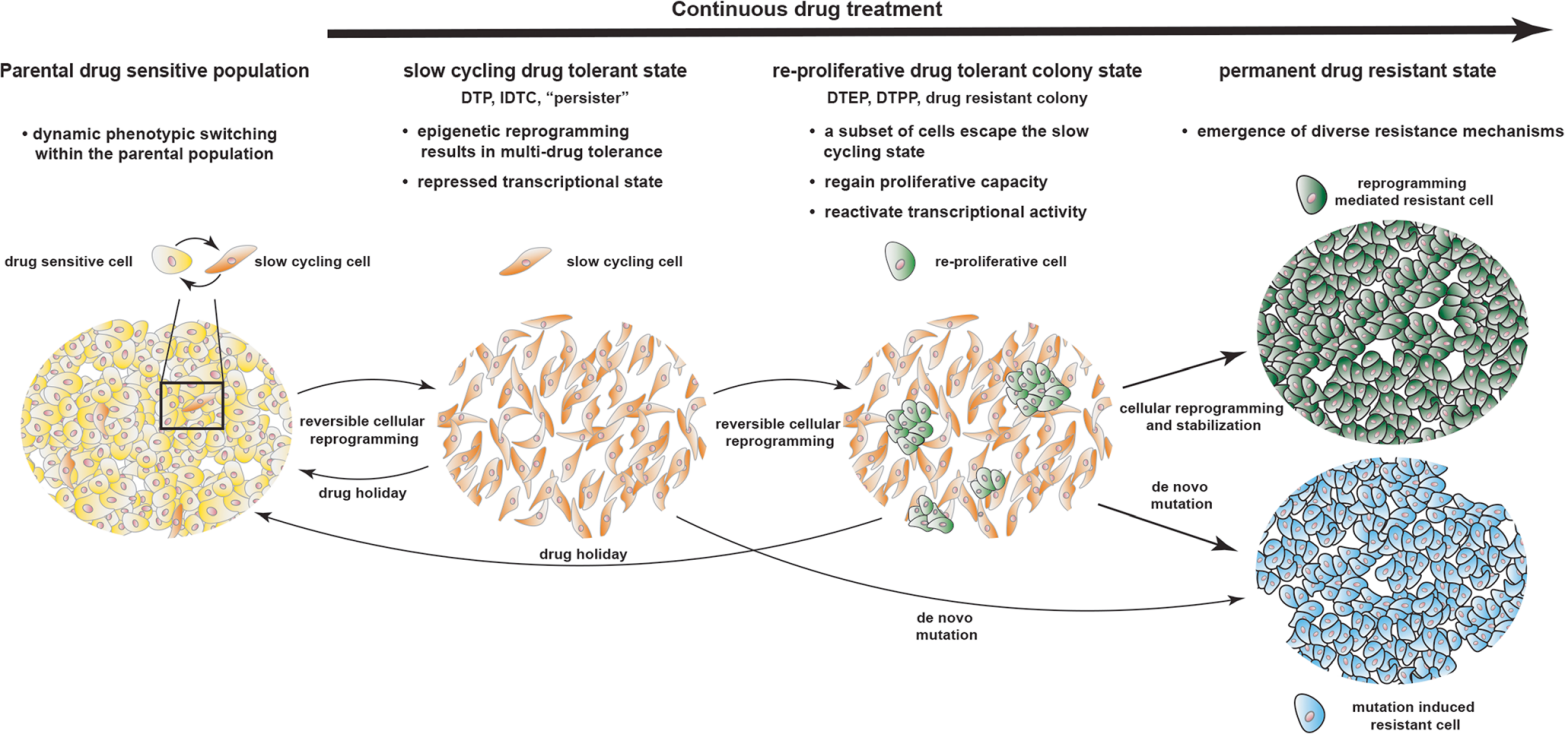
**Proposed model for the development of acquired drug resistance.** A small subset of the parental drug sensitive population undergoes consistent transcriptional reprogramming resulting in phenotypic switching between a proliferative, drug sensitive and a slow cycling drug resistant state. Treatment with targeted therapy or chemotherapy will initially facilitate cellular reprogramming towards the slow cycling drug tolerant phenotype, characterized by repressed transcriptional activity that has been described using different terms in the literature, including drug tolerant persisters (DTP), induced drug tolerant cells (IDTC) or persisters. Continuous drug exposure will eventually lead to reactivation of transcriptional activity in a subset of the slow cycling population, which allows regain of proliferative capacity resulting the the formation of proliferative colonies. This state has been described using terms including drug tolerant expanded persisters (DTEP), drug tolerant proliferating persisters (DTEPP) or drug resistant colonies. These re-proliferative colonies will further stabilize their drug tolerant transcriptional profile to become permanent drug resistant. Alternatively, cells at the slow cycling or colony state acquire de novo mutations during the adaptive transition to become permanently drug resistant

Considering the in vitro time points that are generally described for the drug tolerant slow cycling state (9–14 days) (Guler et al. 2017; Ravindran Menon et al. 2015) and the occurrence of re-proliferating drug tolerant colonies (3–6 weeks) (Shaffer et al. 2017; Song et al. 2017), the current literature supports a state wise transition as the primary response to cytotoxic drugs and targeted inhibitors. Drug sensitive cancer cells adapt by gradually reprogramming their transcriptome. First they respond by reversibly entering a slow cycling multi-drug tolerant state that is characterised by chromatin mediated transcriptional repression and de-differentiation. Continuous drug exposure eventually triggers further cellular reprogramming in a subset of the slow cycling cell population, whereas the cells activate a specific transcriptional profile and re-enter a proliferative state. These re-proliferative colonies are still reversibly drug resistant but eventually stabilize their new transcriptional profile and become permanently drug resistant (Fig. 2).

While development of therapeutic targets often focus on overcomming one of multiple reported mechanisms that mediate resistance to a particular drug, this model offers two novel possible intervention strategies, the slow cycling drug tolerant state and the re-proliferating yet reversible colony state. Successful inhibition of the transition into the slow cycling drug tolerant state could result in the complete erradication of the drug sensitive cancer cell population. Depending on pre-existing tumor heterogeneity, successive or simultaneous drug combinations that exploit vulnerabilities of individual subpopulations with agents that block the adaptive transition into the slow cycling drug tolerant state could therefore hold promise to erradicate tumors.

Another strategy embarks on effective re-sensitization of tumors after disease progression by providing a period of drug holidays (Das et al. 2013; Kurata et al. 2004; Seghers et al. 2012), which might indicate that the re-proliferative colony state is in fact contributing to disease relapse observed in cancer patients. If this proofs to be true, identification of triggers and mechanisms that allow individual cancer cells to escape the drug induced slow cycling state and establish proliferative cellular clusters would be of utmost importance, as these could represent very attractive targets for therapeutic intervention. Inhibiting the transition into the re-proliferative colony state could prevent or delay the emergence of drug resistance and potentially prolong disease remission.

It is important to emphazise that cellular reprogramming mediated resistance and the occurrence of genetic resistance is not necessarily mutually exclusive. Rather the slow cycling drug tolerant population could be seen as a reservouir from which clones with additional aquired muations can arise. (Hata et al. 2016; Ramirez et al. 2016) (Fig. 2). While it remains unclear whether or not blocking cellular reprogramming that allows cancer cells to enter/escape the drug induced slow cycling state has any effect on the frequency of aquired resistance mediating mutations, the very similar features of the drug induced phenotypic transition that is shared by multiple cancer types challeneged with different drugs habors the hope that targeting mechanisms that facillitate drug induced cellular reprogramming could improve a range of well established treatment strategies and aid a broad spectrum of patients suffering from different cancer types.

## References

[CR1] Apostoli AJ, Ailles L (2016). Clonal evolution and tumor-initiating cells: new dimensions in cancer patient treatment. Crit Rev Clin Lab Sci.

[CR2] Balaban NQ, Merrin J, Chait R, Kowalik L, Leibler S (2004). Bacterial persistence as a phenotypic switch. Science.

[CR3] Beretti F, Manni P, Longo C, Argenziano G, Farnetani F, Cesinaro AM, Witkowski AM, De Pol A, Pellacani G (2015). CD271 is expressed in melanomas with more aggressive behaviour, with correlation of characteristic morphology by in vivo reflectance confocal microscopy. Br J Dermatol.

[CR4] Bhang HE, Ruddy DA, Krishnamurthy Radhakrishna V, Caushi JX, Zhao R, Hims MM, Singh AP, Kao I, Rakiec D, Shaw P (2015). Studying clonal dynamics in response to cancer therapy using high-complexity barcoding. Nat Med.

[CR5] Boyd LK, Mao X, Xue L, Lin D, Chaplin T, Kudahetti SC, Stankiewicz E, Yu Y, Beltran L, Shaw G (2012). High-resolution genome-wide copy-number analysis suggests a monoclonal origin of multifocal prostate cancer. Genes Chromosom Cancer.

[CR6] Cabrera MC, Hollingsworth RE, Hurt EM (2015). Cancer stem cell plasticity and tumor hierarchy. World J Stem Cells.

[CR7] Chaffer CL, Brueckmann I, Scheel C, Kaestli AJ, Wiggins PA, Rodrigues LO, Brooks M, Reinhardt F, Su Y, Polyak K (2011). Normal and neoplastic nonstem cells can spontaneously convert to a stem-like state. Proc Natl Acad Sci U S A.

[CR8] Chaffer CL, Marjanovic ND, Lee T, Bell G, Kleer CG, Reinhardt F, D'Alessio AC, Young RA, Weinberg RA (2013). Poised chromatin at the ZEB1 promoter enables breast cancer cell plasticity and enhances tumorigenicity. Cell.

[CR9] Das TM, Salangsang F, Landman AS, Sellers WR, Pryer NK, Levesque MP, Dummer R, McMahon M, Stuart DD (2013). Modelling vemurafenib resistance in melanoma reveals a strategy to forestall drug resistance. Nature.

[CR10] Fidler IJ (1978). Tumor heterogeneity and the biology of cancer invasion and metastasis. Cancer Res.

[CR11] Gay, L., Baker, A.M., and Graham, T.A. (2016). Tumour Cell Heterogeneity. F1000Res 5 10.12688/f1000research.7210.1 PMC4776671 26973786

[CR12] Greulich H, Chen TH, Feng W, Janne PA, Alvarez JV, Zappaterra M, Bulmer SE, Frank DA, Hahn WC, Sellers WR (2005). Oncogenic transformation by inhibitor-sensitive and -resistant EGFR mutants. PLoS Med.

[CR13] Guler GD, Tindell CA, Pitti R, Wilson C, Nichols K, KaiWai Cheung T, Kim HJ, Wongchenko M, Yan Y, Haley B (2017). Repression of stress-induced LINE-1 expression protects cancer cell subpopulations from lethal drug exposure. Cancer Cell.

[CR14] Gupta PB, Fillmore CM, Jiang G, Shapira SD, Tao K, Kuperwasser C, Lander ES (2011). Stochastic state transitions give rise to phenotypic equilibrium in populations of cancer cells. Cell.

[CR15] Hanahan D (2014). Rethinking the war on cancer. Lancet.

[CR16] Hata AN, Niederst MJ, Archibald HL, Gomez-Caraballo M, Siddiqui FM, Mulvey HE, Maruvka YE, Ji F, Bhang HE, Krishnamurthy Radhakrishna V (2016). Tumor cells can follow distinct evolutionary paths to become resistant to epidermal growth factor receptor inhibition. Nat Med.

[CR17] Hauschild A, Grob JJ, Demidov LV, Jouary T, Gutzmer R, Millward M, Rutkowski P, Blank CU, Miller WH, Kaempgen E (2012). Dabrafenib in BRAF-mutated metastatic melanoma: a multicentre, open-label, phase 3 randomised controlled trial. Lancet.

[CR18] Hoek KS, Eichhoff OM, Schlegel NC, Dobbeling U, Kobert N, Schaerer L, Hemmi S, Dummer R (2008). In vivo switching of human melanoma cells between proliferative and invasive states. Cancer Res.

[CR19] Holden DW (2015). Microbiology. Persisters unmasked. Science.

[CR20] Holohan C, Van Schaeybroeck S, Longley DB, Johnston PG (2013). Cancer drug resistance: an evolving paradigm. Nat Rev Cancer.

[CR21] Hyman DM, Puzanov I, Subbiah V, Faris JE, Chau I, Blay JY, Wolf J, Raje NS, Diamond EL, Hollebecque A (2015). Vemurafenib in multiple nonmelanoma cancers with BRAF V600 mutations. N Engl J Med.

[CR22] Kurata T, Tamura K, Kaneda H, Nogami T, Uejima H, Asai Go G, Nakagawa K, Fukuoka M (2004). Effect of re-treatment with gefitinib ('Iressa', ZD1839) after acquisition of resistance. Ann Oncol.

[CR23] Lee Y, Lee GK, Lee YS, Zhang W, Hwang JA, Nam BH, Kim SH, Kim JH, Yun T, Han JY (2014). Clinical outcome according to the level of preexisting epidermal growth factor receptor T790M mutation in patients with lung cancer harboring sensitive epidermal growth factor receptor mutations. Cancer.

[CR24] Liao BC, Lin CC, Lee JH, Yang JC (2016). Update on recent preclinical and clinical studies of T790M mutant-specific irreversible epidermal growth factor receptor tyrosine kinase inhibitors. J Biomed Sci.

[CR25] Liau BB, Sievers C, Donohue LK, Gillespie SM, Flavahan WA, Miller TE, Venteicher AS, Hebert CH, Carey CD, Rodig SJ (2017). Adaptive chromatin remodeling drives glioblastoma stem cell plasticity and drug tolerance. Cell Stem Cell.

[CR26] Lin J, Goto Y, Murata H, Sakaizawa K, Uchiyama A, Saida T, Takata M (2011). Polyclonality of BRAF mutations in primary melanoma and the selection of mutant alleles during progression. Br J Cancer.

[CR27] Marusyk A, Polyak K (2010). Tumor heterogeneity: causes and consequences. Biochim Biophys Acta.

[CR28] Marusyk A, Almendro V, Polyak K (2012). Intra-tumour heterogeneity: a looking glass for cancer?. Nat Rev Cancer.

[CR29] McGranahan N, Swanton C (2017). Clonal heterogeneity and tumor evolution: past, present, and the future. Cell.

[CR30] Mok TS, Wu YL, Thongprasert S, Yang CH, Chu DT, Saijo N, Sunpaweravong P, Han B, Margono B, Ichinose Y (2009). Gefitinib or carboplatin-paclitaxel in pulmonary adenocarcinoma. N Engl J Med.

[CR31] Mok TS, Wu YL, Ahn MJ, Garassino MC, Kim HR, Ramalingam SS, Shepherd FA, He Y, Akamatsu H, Theelen WS (2017). Osimertinib or platinum-Pemetrexed in EGFR T790M-positive lung cancer. N Engl J Med.

[CR32] Naidoo J, Sima CS, Rodriguez K, Busby N, Nafa K, Ladanyi M, Riely GJ, Kris MG, Arcila ME, Yu HA (2015). Epidermal growth factor receptor exon 20 insertions in advanced lung adenocarcinomas: clinical outcomes and response to erlotinib. Cancer.

[CR33] Prahallad A, Sun C, Huang S, Di Nicolantonio F, Salazar R, Zecchin D, Beijersbergen RL, Bardelli A, Bernards R (2012). Unresponsiveness of colon cancer to BRAF(V600E) inhibition through feedback activation of EGFR. Nature.

[CR34] Quintana E, Shackleton M, Foster HR, Fullen DR, Sabel MS, Johnson TM, Morrison SJ (2010). Phenotypic heterogeneity among tumorigenic melanoma cells from patients that is reversible and not hierarchically organized. Cancer Cell.

[CR35] Ramirez M, Rajaram S, Steininger RJ, Osipchuk D, Roth MA, Morinishi LS, Evans L, Ji W, Hsu CH, Thurley K (2016). Diverse drug-resistance mechanisms can emerge from drug-tolerant cancer persister cells. Nat Commun.

[CR36] Ravindran Menon D, Das S, Krepler C, Vultur A, Rinner B, Schauer S, Kashofer K, Wagner K, Zhang G, Bonyadi Rad E (2015). A stress-induced early innate response causes multidrug tolerance in melanoma. Oncogene.

[CR37] Roesch A, Fukunaga-Kalabis M, Schmidt EC, Zabierowski SE, Brafford PA, Vultur A, Basu D, Gimotty P, Vogt T, Herlyn M (2010). A temporarily distinct subpopulation of slow-cycling melanoma cells is required for continuous tumor growth. Cell.

[CR38] Roesch A, Vultur A, Bogeski I, Wang H, Zimmermann KM, Speicher D, Korbel C, Laschke MW, Gimotty PA, Philipp SE (2013). Overcoming intrinsic multidrug resistance in melanoma by blocking the mitochondrial respiratory chain of slow-cycling JARID1B(high) cells. Cancer Cell.

[CR39] Rycaj K, Tang DG (2015). Cell-of-origin of Cancer versus cancer stem cells: assays and interpretations. Cancer Res.

[CR40] Seghers AC, Wilgenhof S, Lebbe C, Neyns B (2012). Successful rechallenge in two patients with BRAF-V600-mutant melanoma who experienced previous progression during treatment with a selective BRAF inhibitor. Melanoma Res.

[CR41] Sensi M, Nicolini G, Petti C, Bersani I, Lozupone F, Molla A, Vegetti C, Nonaka D, Mortarini R, Parmiani G (2006). Mutually exclusive NRASQ61R and BRAFV600E mutations at the single-cell level in the same human melanoma. Oncogene.

[CR42] Shackleton M, Quintana E, Fearon ER, Morrison SJ (2009). Heterogeneity in cancer: cancer stem cells versus clonal evolution. Cell.

[CR43] Shaffer SM, Dunagin MC, Torborg SR, Torre EA, Emert B, Krepler C, Beqiri M, Sproesser K, Brafford PA, Xiao M (2017). Rare cell variability and drug-induced reprogramming as a mode of cancer drug resistance. Nature.

[CR44] Sharma SV, Lee DY, Li B, Quinlan MP, Takahashi F, Maheswaran S, McDermott U, Azizian N, Zou L, Fischbach MA (2010). A chromatin-mediated reversible drug-tolerant state in cancer cell subpopulations. Cell.

[CR45] Sharma P, Hu-Lieskovan S, Wargo JA, Ribas A (2017). Primary, adaptive, and acquired resistance to cancer immunotherapy. Cell.

[CR46] Shi H, Hugo W, Kong X, Hong A, Koya RC, Moriceau G, Chodon T, Guo R, Johnson DB, Dahlman KB (2014). Acquired resistance and clonal evolution in melanoma during BRAF inhibitor therapy. Cancer Discov.

[CR47] Slingluff CL, Colella TA, Thompson L, Graham DD, Skipper JC, Caldwell J, Brinckerhoff L, Kittlesen DJ, Deacon DH, Oei C (2000). Melanomas with concordant loss of multiple melanocytic differentiation proteins: immune escape that may be overcome by targeting unique or undefined antigens. Cancer Immunol Immunother.

[CR48] Song C, Piva M, Sun L, Hong A, Moriceau G, Kong X, Zhang H, Lomeli S, Qian J, Yu CC, et al (2017) Recurrent tumor cell-intrinsic and -extrinsic alterations during MAPKi-induced melanoma regression and early adaptation. Cancer Discov 7:1248–1265 10.1158/2159-8290.CD-17-0401 PMC6668729 28864476

[CR49] Thress KS, Paweletz CP, Felip E, Cho BC, Stetson D, Dougherty B, Lai Z, Markovets A, Vivancos A, Kuang Y (2015). Acquired EGFR C797S mutation mediates resistance to AZD9291 in non-small cell lung cancer harboring EGFR T790M. Nat Med.

[CR50] Ugurel S, Rohmel J, Ascierto PA, Flaherty KT, Grob JJ, Hauschild A, Larkin J, Long GV, Lorigan P, McArthur GA (2016). Survival of patients with advanced metastatic melanoma: the impact of novel therapies. Eur J Cancer.

[CR51] Verfaillie A, Imrichova H, Atak ZK, Dewaele M, Rambow F, Hulselmans G, Christiaens V, Svetlichnyy D, Luciani F, Van den Mooter L (2015). Decoding the regulatory landscape of melanoma reveals TEADS as regulators of the invasive cell state. Nat Commun.

[CR52] Vlashi E, Pajonk F (2015). Cancer stem cells, cancer cell plasticity and radiation therapy. Semin Cancer Biol.

[CR53] Wang J, Wang B, Chu H, Yao Y (2016). Intrinsic resistance to EGFR tyrosine kinase inhibitors in advanced non-small-cell lung cancer with activating EGFR mutations. Onco Targets Ther.

[CR54] Yancovitz M, Litterman A, Yoon J, Ng E, Shapiro RL, Berman RS, Pavlick AC, Darvishian F, Christos P, Mazumdar M et al (2012) Intra- and inter-tumor heterogeneity of BRAF(V600E))mutations in primary and metastatic melanoma. PLoS One 7:e29336 10.1371/journal.pone.0029336 PMC3250426 22235286

[CR55] Yu HA, Arcila ME, Rekhtman N, Sima CS, Zakowski MF, Pao W, Kris MG, Miller VA, Ladanyi M, Riely GJ (2013). Analysis of tumor specimens at the time of acquired resistance to EGFR-TKI therapy in 155 patients with EGFR-mutant lung cancers. Clinical cancer research: an official journal of the American Association for Cancer Research.

